# An Online, Self-Directed Curriculum of Core Research Concepts and Skills

**DOI:** 10.15766/mep_2374-8265.10732

**Published:** 2018-07-27

**Authors:** Adrienne A. Williams, Shana O. Ntiri

**Affiliations:** 1 Associate Professor, Department of Family Medicine, University of Illinois College of Medicine at Chicago; 2 Assistant Professor, Department of Family and Community Medicine, University of Maryland School of Medicine

**Keywords:** Technology, Curriculum, Residency, Family Medicine, Research, Online, Scholarly

## Abstract

**Introduction:**

Existing scholarly curricula often underemphasize basic research skills and do not address the individual learning needs of residents, whose level of prior exposure to research concepts varies widely. A supplemental educational experience was developed to address educational gaps in a family medicine residency curriculum, including systematic exploration and interpretation of the medical literature, development and exploration of clinically pertinent questions, and development of residents' written communication skills.

**Methods:**

A 2-week, online, self-directed research curriculum was developed. The five-module curriculum included (I) Research Methods and Data Analysis, (II) Article Review, (III) Board Review, (IV) Literature Search, and (V) Literature Review and Proposal. Two years after implementation, residents who completed the curriculum were surveyed to assess the overall rotation and its success in meeting learning objectives.

**Results:**

Eighteen residents completed the new rotation and demonstrated objectives through assignment completion and review. Additionally, residents reported improved skills on all objectives and were satisfied with the new curriculum and its self-led, online format. Those planning to do research after graduation were more likely to report several benefits from the rotation, including learning more about data analyses and being more likely to complete a future scholarly project.

**Discussion:**

An online, self-directed curriculum can provide a feasible and effective educational approach to efficient use of faculty and resident time, allowing time to be focused on resident-specific knowledge gaps and learning needs, rather than presenting all learning material uniformly. The online, accessible format aligned with residents' existing reliance on the internet as a primary information source.

## Educational Objectives

By the end of this curriculum, learners will be able to:

1. Apply critical reading skills to determine study validity and interpret study results in the medical literature.
2. Demonstrate ability to critically evaluate research and medical literature.
3. Identify strengths and weaknesses in study designs.
4. Practice calculating and interpreting basic statistics.
5. Apply basic statistical concepts to interpreting the medical literature.
6. Synthesize knowledge gained in the curriculum to develop a research question and methods to evaluate the question.
7. Develop writing skills through preparation of a project proposal.

## Introduction

Since 2015, the Accreditation Council for Graduate Medical Education (ACGME) has required all 27 of its hospital-based medical and surgical specialty residencies to include scholarly activity as a component of their program curricula and to allocate educational resources to support residents' participation in scholarly activities.^1^ The ACGME Program Requirements for Graduate Medical Education in Family Medicine specify that the scholarly activity curriculum should enhance residents' understanding of research—including how research is conducted, evaluated, and used to inform patient care.^2^ The ACGME's Family Medicine Review Committee began requiring resident participation in scholarly activity for residencies in 2006.^3^ Crawford and Seehusen found that 2 years after implementation of the requirement, only 42.8% of the family medicine residencies surveyed had altered their program requirements, and 23.4% of programs did not have a scholarly activity curriculum in place.^3^

The multifaceted approaches residencies have used to fulfill the ACGME requirements incorporate journal clubs, resident/faculty collaboration, and individual or team-based research and quality improvement projects.^4,5^ At the University of Maryland's Department of Family and Community Medicine (DFCM)—an urban, academic department of family medicine—the initial scholarly activity curriculum implemented in response to the ACGME requirements had components similar to those seen at other programs. The longitudinal curriculum began during residents' first year (PGY-1), with faculty lectures on scholarly activity topics and an introduction to departmental research activities. During the second year (PGY-2), residents participated in monthly journal clubs, led by a PGY-2 resident who reviewed and presented a faculty-approved article to residents and faculty. In the third year (PGY-3), residents worked individually or in pairs on a quality improvement or research project, which culminated with a brief oral presentation of their project at a departmental research day at the end of each academic year.

While this curriculum integrated commonly utilized curricular components, there were a number of educational gaps. The curriculum did not account for the fact that residents entered the program with varying prior knowledge of and exposure to scholarly activity. The curriculum did not teach residents how to systematically explore and interpret the medical literature for the purpose of developing and answering clinically pertinent questions. Additionally, the initial curriculum did not incorporate opportunities for residents to develop their written communication skills. To address these educational gaps in our residency, the DFCM developed and implemented a 2-week research rotation for all second-year residents to supplement the residency program's existing scholarly activity curriculum.

### Curriculum Goals

The research faculty met to discuss common areas of resident weakness, which were identified through faculty discussions of areas in which residents regularly needed assistance during existing scholarly activities. Common problem areas included interpreting the data analysis section of journal club articles and difficulties with the application of the medical literature. As noted in other residencies, residents were accustomed to utilizing non-evidence-based search engines to answer point-of-care clinical questions, and many residents were unsure of how and when it was appropriate to apply recommendations from the literature to specific clinical situations.^6^ Furthermore, as PGY-3 residents developed their projects, many were inexperienced with developing an appropriate question and approach to answer their question. Resident feedback on the existing scholarly curriculum was also examined, and common themes were noted. Informal resident feedback revealed that residents were not comfortable with understanding research methods, data analysis, or basic statistical concepts. Residents did not see how the existing curriculum was applicable to their future clinical practice or in preparing for their board exams. Additionally, in contrast to their usual learning style, the existing scholarly curriculum did not incorporate online resources. These key problem areas were developed into learning objectives.

It was determined that residents needed more opportunity earlier in their training to learn the fundamentals of scholarly activity. Addressing this need was initially seen as challenging because identifying and addressing each resident's individual needs for scholarly knowledge and skills would require substantial faculty time. Additionally, the number of faculty who felt comfortable enough with their own scholarly activity knowledge to provide resident education in this area was limited. Thus, any proposed solution would also have to take this into account.

We conducted a literature search, which did not reveal any curricula that addressed all of our specific needs. We therefore decided to develop a curriculum that fit with our learning needs, available resources, and the learning preferences of our residents. A self-led curriculum was selected so that residents had the ability to tailor the rotation to their learning needs by spending more time on a topic that they were not familiar with and less time on a concept they understood well. The online format made it easy for residents to access the curriculum at any time and from any location and also created a resource residents could return to for information even after residency. Additionally, we aimed to develop a curriculum that increased resident satisfaction, as satisfaction with a curriculum could impact future interest.

A list of seven objectives was developed to address the identified needs. The new curriculum would be designed to help residents (1) apply critical reading skills to determine study validity and interpret study results in the medical literature, (2) demonstrate ability to critically evaluate research and medical literature, (3) identify strengths and weaknesses in study designs, (4) practice calculating and interpreting basic statistics, (5) apply basic statistical concepts to interpreting the medical literature, (6) synthesize knowledge gained in the curriculum to develop a research question and methods to evaluate the question, and (7) develop writing skills through preparation of a project proposal. Each objective would be demonstrated through online assignments and meeting with faculty.

## Methods

### Rotation Structure

#### Schedule

The new 2-week research rotation was required and employed a self-directed, online, modular research curriculum that residents completed during their PGY-2 year. All curriculum materials were provided online through the learning management system (LMS) at our university (Blackboard Learn, Release 9.1), and instructions were summarized in a handout (Appendix A). During the 2-week rotation, residents were assigned 4 half-days of clinic and 2 half-days of didactics, leaving 8 half-days dedicated to the curriculum. PGY-2 residents were assigned to the research rotation throughout the academic year such that only one resident completed the new curriculum at a time.

#### Supervision

In addition to the online sections of the curriculum, residents also had three individual meetings with faculty. These meetings provided orientation, teaching, and individual feedback to the resident. To increase schedule flexibility and decrease faculty time burden, residents could meet with any of the faculty trained to supervise this rotation for each of the three meetings. Thus, residents could meet with the same faculty member for all three supervision meetings or meet with different faculty members for each meeting. Each meeting was scheduled based on availability of both the resident and the faculty member. Supervision typically required 2.5–3 hours of faculty time: 15–30 minutes for the premeeting and 1 hour for each of the other two meetings. Additional time may have been needed to review resident responses and revised proposal drafts. After the rotation, each faculty member would complete a resident evaluation applicable to his or her meeting(s) with the resident.

#### Faculty preparation

Three faculty members worked with residents during the rotation. The faculty members' level of research experience varied from informal exposure to graduate-level training in statistics and research methods. Rotation faculty were expected to read through the documents provided to residents during the rotation, with special attention to Module II articles. Faculty met annually to review the curriculum materials, as well as key concepts and discussion points for each module. An answer key and a list of critiques for Module II articles were provided to faculty and reviewed annually to ensure all faculty had a thorough understanding of rotation content and teaching points. Instructors also met at the beginning of each academic year to discuss expectations of learners for each component of the curriculum, including what factors would constitute excellence for each category. Rubrics were not provided to the learners as a component of the curriculum. However, during the orientation meeting, residents were provided with a rotation overview that outlined expectations for all aspects of the curriculum that featured specific guidance and references for scientific writing.

### Curriculum Components

The five-module curriculum included (I) Research Methods and Data Analysis, (II) Article Review, (III) Board Review, (IV) Literature Search, and (V) Literature Review and Proposal. Each of the five modules was designed to meet different objectives for the rotation (Table 1).

**Table 1.** Curriculum Modules Addressing Each Objective

Curriculum Objective	Curriculum Module				
	I	II	III	IV	V
1. Apply critical reading skills to determine study validity and interpret study results in the medical literature.	X	X		X	
2. Demonstrate ability to critically evaluate research and medical literature.	X	X		X	
3. Identify strengths and weaknesses in study designs.	X	X			
4. Practice calculating and interpreting basic statistics.	X	X	X		
5. Apply basic statistical concepts to interpreting the medical literature.	X	X	X		
6. Synthesize knowledge gained in the curriculum to develop a research question and methods to evaluate the question.	X	X		X	X
7. Develop writing skills through preparation of a project proposal.		X		X	X

#### Faculty premeeting

Prior to the scheduled 2-week rotation, residents met with a rotation faculty for orientation to the rotation. This meeting typically lasted 15–30 minutes, and the meeting objective was to review rotation expectations and format of the rotation, since the structure was different from other residency rotations. The faculty ensured that residents knew how to access the online curriculum, as well as how to enter and check answers to review questions. Requirements for the rotation were reviewed, including due dates and how self-led time could be used.

#### Module I—Research Methods and Data Analysis

Module I comprised a series of readings to enhance knowledge and understanding of scientific literature, research methods, data analysis, and writing skills (Table 2). These background readings provided the knowledge base and references that were used to complete the remaining four modules.

**Table 2.** Assigned Readings for the Curriculum

Readings	Topics Covered	Curriculum Objectives^a^
Module I		
Oxman AD, Sackett DL, Guyatt GH, et al. Users' guides to the medical literature: I. How to get started. *JAMA*. 1993;270(17):2093–2095.	Forming a question and finding references	1, 3
Guyatt GH, Sackett DL, Cook DJ, et al. Users' guides to the medical literature: II. How to use an article about therapy or prevention. A. Are the results of the study valid? *JAMA*. 1993;270(21):2598–2601.	Determining study validity (prevention and treatment)	1, 3, 5
Guyatt GH, Sackett DL, Cook DJ, et al. Users' guides to the medical literature: II. How to use an article about therapy or prevention. B. What were the results and will they help me in caring for my patients? *JAMA*. 1994;271(1):59–63.	Interpreting study results (prevention and treatment)	1, 2, 5
Jaeschke R, Guyatt G, Sackett DL, et al. Users' guides to the medical literature: III. How to use an article about a diagnostic test. A. Are the results of the study valid? *JAMA*. 1994;271(5):389–391.	Determining study validity (diagnostic tests)	1, 2, 3, 5
Jaeschke R, Guyatt GH, Sackett DL, et al. Users' guides to the medical literature: III. How to use an article about a diagnostic test. B. What are the results and will they help me in caring for my patients? *JAMA*. 1994;271(9):703–707.	Interpreting study results (diagnostic tests)	1, 2, 3, 5
Laupacis A, Wells G, Richardson WS, et al. Users' guides to the medical literature: V. How to use an article about prognosis. *JAMA.* 1994;272(3):234–237.	Applying study results (prognosis)	1, 3
Bewick V, Cheek L, Ball J. Statistics review 13: receiver operating characteristic curves. *Crit Care*. 2004;8(6):508–512.	Sensitivity, specificity, positive/negative predictive values, likelihood ratios, receiver operating characteristic curves	1, 2, 3, 4, 5
Matthews JR, Matthews RW. Follow standard structure. In: *Successful Scientific Writing: A Step-by-Step Guide for the Biological and Medical Sciences*. 3rd ed. Cambridge, England: Cambridge University Press; 2008:42–48.	Structure of research articles	6, 7
Bem DJ. Writing the empirical journal article. In: Darley JM, Zanna MP, Roediger HL III, eds. *The Compleat Academic: A Career Guide.* 2nd ed. Washington, DC: American Psychological Association; 2004:185–219.	Scientific and concise writing	6, 7
Module II		
Lynn R. Race differences in sexual behavior and their demographic implications. *Popul Environ*. 2000;22(1):73–81.	Theory, methods, statistical/clinical significance, data-based conclusions	1, 2, 3
MacMahon B, Yen S, Trichopoulos D, Warren K, Nardi G. Coffee and cancer of the pancreas. *N Engl J Med*. 1981;304(11):630–633.	*p* values	1, 2, 3, 5
Kulasingam SL, Hughes JP, Kiviat NB, et al. Evaluation of human papillomavirus testing in primary screening for cervical abnormalities: comparison of sensitivity, specificity, and frequency of referral. *JAMA*. 2002;288(14):1749–1757.	Sensitivity, specificity, positive and negative predictive values	1, 4, 5
Ferreira FL, Bota DP, Bross A, Mélot C, Vincent J-L. Serial evaluation of the SOFA score to predict outcome in critically ill patients. *JAMA*. 2001;286(14):1754–1758.	Receiver operating characteristic curves	1, 4, 5

a See Table 1 for curriculum objectives.

Residents were first assigned several readings from the “Users' Guides to the Medical Literature” series of articles.^7–12^ Residents were provided online access to the entire series; however, required readings consisted only of sections I-III and V. These sections provided a basic introduction on how to formulate an appropriate question, conduct a thorough literature search, critically review the literature, and come to a conclusion based on what was read. These readings were selected to help residents better search and understand the medical literature and apply findings to clinical cases and scholarly projects.

Residents were also provided with a one-page document (Appendix B), written for the rotation, that briefly reviewed concepts on how to determine the validity of a study. These concepts included variable definitions, measurement of defined variables, rationality of conclusions, and the interpretation of *p* values.

Next, residents read “Statistics Review 13: Receiver Operating Characteristic Curves,”^13^ a brief, five-page article that provided a review of sensitivity, specificity, positive predictive value, negative predictive value, likelihood ratios, and receiver operating characteristic (ROC) curves. The article presented the basic information about these statistical measures, including their calculation and interpretation. This article was selected in response to resident feedback regarding concerns about understanding basic statistical concepts and served as a useful resource to reference while reading the medical literature and preparing for board exams.

For the last section of Module I, residents were asked to read two publications on how to write. The first reading was a selection concerning the standard structure of research literature from Matthews and Matthews' *Successful Scientific Writing: A Step-by-Step Guide for the Biological and Medical Sciences.*^14^ This selection was meant to prepare residents for reading the literature and to help them structure their own writing. The second selection was from Bem's chapter in *The Compleat Academic: A Career Guide*.^15^ This chapter provided guidance on preparing to write, structuring what to write, and how to write in a clear, concise manner. It was selected to aid residents with both scholarly and clinical writing when working on the core competency of communication.

#### Module II—Article Review

Module II required residents to apply the skills learned in Module I, critically reading and responding to questions for each of four journal articles (Table 2). Questions and sample answers for each of the four articles described below are available in Appendices C and D.

The first article, “Race Differences in Sexual Behavior and Their Demographic Implications”^16^—a relatively recent article from a peer-reviewed journal—had numerous flaws in its theory application, methods, data interpretation, and conclusions. This article was also selected because, for many residents, its theory and conclusions would be emotionally inflammatory. Thus, residents were highly motivated to find as many limitations with the study as possible. During faculty discussion 1 (see below), it was emphasized that all literature should be read with the same level of enthusiasm and rigor for critical analysis as was elicited by an article that residents felt motivated to disprove. After reading this article, residents submitted their critical analysis of each section (Background, Methods, Results, Discussion) online.

The second article, “Coffee and Cancer of the Pancreas,”^17^ was selected to demonstrate the meaning of *p* values and to illustrate that even well-designed studies may result in statistically significant findings solely by chance. This article was selected because it came from a reputable journal and included findings that would impact a physician's practice but that were not able to be replicated. After reading this article, residents were presented with the apparent conflict that although this well-designed study in a reputable journal concluded there was a strong association between pancreatic cancer and coffee consumption, the American Cancer Society no longer considers coffee as a significant risk factor for pancreatic cancer. The residents then responded to questions in the LMS about factors that could lead to questioning the conclusions of this study.

To practice calculating and interpreting statistical findings, residents read “Evaluation of Human Papillomavirus Testing in Primary Screening for Cervical Abnormalities: Comparison of Sensitivity, Specificity, and Frequency of Referral”^18^ and “Serial Evaluation of the SOFA Score to Predict Outcome in Critically Ill Patients”^19^ and used data from these articles to respond to questions through the LMS. The first of these two articles was selected because it contained raw numbers that residents could use to calculate sensitivity, specificity, positive predictive value, and negative predictive value. The second article was chosen because it contained multiple ROC curves for the residents to practice interpreting. Residents had the opportunity to calculate and interpret sensitivity, specificity, positive predictive value, negative predictive value, and ROC curves using data from the assigned articles. Responses to multiple-choice questions were scored automatically through the LMS website, providing residents with immediate feedback on their work.

#### Module III—Board Review

Module III allowed residents to practice using statistical knowledge by answering 20 board review–type questions posted on the LMS. The LMS automatically scored the multiple-choice questions and provided feedback for each question. This module allowed residents to identify key concepts that they needed to review and thematic areas to discuss during their one-on-one faculty meetings. Residents were able to correct and resubmit their responses in order to monitor their improvement in knowledge. The LMS allowed faculty to see whether residents required more than one attempt to correctly answer questions. If it was found that residents needed more than one attempt on certain questions, those questions were further reviewed during the faculty discussion (see below).

#### Faculty discussion 1

After completing the first three modules, residents had a 1-hour discussion with one of the rotation faculty. During this discussion, the faculty reviewed all answers submitted online with the resident, ensured that the resident had an adequate understanding of how to critically read articles, and further discussed concepts that the resident did not thoroughly understand on his or her own.

#### Module IV—Literature Search

Module IV guided residents through developing a clinical or research question and completing a literature search. Through online instructions, residents learned about various databases and how to differentiate between appropriate (i.e., peer-reviewed journal articles) and inappropriate (e.g., commercial websites, general search engines, Wikipedia) sources of information. Residents developed a question and began a literature search.

#### Module V—Literature Review and Proposal

Module V led the resident through writing a literature review and a project proposal. Residents were responsible for writing an introduction and methods section for their proposed project and citing at least 10 appropriate resources for their proposal. The project proposal could serve as the foundation for the scholarly project that residents were to complete during their PGY-3 year. By writing a project proposal during the PGY-2 year, residents had adequate time to develop a feasible, well-thought-out project with measurable outcomes.

#### Faculty discussion 2

Residents met with a faculty member for a second 1-hour discussion. The faculty reviewed the first draft of the resident's literature review and methods section. Residents were provided with feedback on their writing and the empirical soundness of proposed methods for answering their clinical/research question. After this second discussion, residents revised and resubmitted their literature review and project proposal via email to the faculty member they had met with for the second discussion. Further meetings to discuss this proposal were scheduled as deemed necessary by either the faculty or the resident.

### Evaluation of Curriculum

Since demonstrating each of the learning objectives was required for residents to pass the rotation, we decided to further evaluate the residents' experience of the curriculum. After the curriculum had been implemented for 2 years, all 18 residents who had completed the rotation in the preceding 24 months were invited to fill out an anonymous online survey to evaluate the rotation. The survey was designed to evaluate the success of the rotation in addressing the original identified needs. The objectives of the survey were to assess resident satisfaction with the overall rotation, resident comfort with achieving learning objectives, and impact of the rotation on future planned participation in research and/or scholarly activities.

All residents were emailed a link to the survey. As a maximum of one resident completed the rotation per month, surveying all the residents at once ensured that responses were not easily identifiable. Residents were given approximately 4 weeks to complete the survey and were sent two email reminders during that time.

The survey included two questions about overall satisfaction with the rotation and the self-directed, online curriculum structure, each scored on a 5-point Likert scale ranging from *very unsatisfied* to *very satisfied*; 11 questions about the rotation's learning objectives, scored on a 5-point Likert scale ranging from *strongly disagree* to *strongly agree*; and six dichotomous items about plans to engage in future research and/or scholarly activities. Data were analyzed using SPSS software, version 22. The study was deemed exempt by the institutional review board at the University of Maryland, Baltimore.

## Results

### Overall Rotation Outcomes

The incorporation of this 2-week rotation into our longitudinal scholarly activity curriculum provided a feasible way to increase the exposure of residents to research. The online format provided a resource that residents became very familiar with during their rotation and could refer back to any time they had a research question. The self-led structure of the rotation enabled adequate faculty supervision of the rotation without overburdening faculty. This format also enabled residents to individually tailor the curriculum to their specific learning needs by spending more or less time on a particular concept dependent on their baseline knowledge of the topic. The format enabled both faculty and residents to identify and address gaps in research skills early on. Because residents completed this rotation in their PGY-2 year, it gave them more opportunity to practice application of evidence to clinical scenarios earlier on in their training. Additionally, the rotation provided residents with the opportunity to identify a topic of interest and to develop and refine the question they desired to investigate for their PGY-3 scholarly activity project prior to the official start of that project.

### Resident Evaluation Survey

Sixteen of the 18 eligible residents (89%) completed the survey. Sixty-nine percent (*n* = 11) indicated that they were satisfied or very satisfied with the overall rotation. None indicated that they were unsatisfied or very unsatisfied with the overall rotation. Residents felt positively about the online/self-led structure of the rotation, with 81% (*n* = 13) indicating that they were satisfied or very satisfied with this aspect of the curriculum.

Residents felt that the rotation successfully achieved its learning objectives. Residents reported that as a result of the curriculum, they felt more confident in reading/understanding the medical literature, were better able to critically appraise medical research literature, learned more about research methods and data analysis, had a better understanding of how to interpret basic statistical concepts, felt more comfortable applying information from the medical literature to their clinical practice, developed an understanding of the process for preparing a research proposal, had improved writing skills, and were more likely to complete a scholarly project in the future (Table 3).

**Table 3.** Resident Evaluation of Research Curriculum (*N* = 16)

Item	Strongly Agree	Agree	Neutral	Disagree	Strongly Disagree
Curriculum objectives					
As a result of the rotation:					
I understand the process for preparing a research proposal.	1 (7%)	11 (73%)	3 (20%)	0 (0%)	0 (0%)
I learned more about research methods.	0 (0%)	12 (75%)	3 (19%)	1 (6%)	0 (0%)
I am better able to critically appraise research and literature.	1 (6%)	10 (63%)	3 (19%)	2 (12%)	0 (0%)
I learned more about data analysis.	0 (0%)	10 (67%)	4 (27%)	1 (6%)	0 (0%)
I have improved my scholarly or research writing skills.	1 (6%)	9 (56%)	6 (38%)	0 (0%)	0 (0%)
I am more likely to complete a scholarly project in the future.	1 (6%)	8 (50%)	5 (31%)	2 (13%)	0 (0%)
I have a better understanding of how to interpret basic statistical concepts, such as PPV, NPV, ROC curve.	1 (6%)	8 (50%)	5 (31%)	2 (13%)	0 (0%)
I feel more comfortable applying information from the medical literature to my practice.	0 (0%)	11 (69%)	2 (12%)	3 (19%)	0 (0%)
I feel more confident in reading/understanding the medical literature.	0 (0%)	11 (69%)	1 (6%)	3 (19%)	1 (6%)
Curriculum structure					
I found the protected time to complete this rotation helpful.	3 (19%)	11 (69%)	2 (12%)	0 (0%)	0 (0%)
There was sufficient protected time for adequate self-directed learning.	3 (19%)	7 (44%)	4 (25%)	2 (12%)	0 (0%)

Abbreviations: NPV, negative predictive value; PPV, positive predictive value; ROC, receiver operating characteristic.

Over half of respondents (*n* = 9, 56%) said that after residency, they were likely to perform quality improvement, teach or precept medical learners, or collaborate on a research project. About one-third (*n* = 5, 31%) thought they might give a scholarly presentation after residency. One-quarter (*n* = 4, 25%) stated that they were likely to initiate a research project, and 19% (*n* = 3) thought they were likely to pursue grant funding. Only one respondent (6%) indicated not being likely to do any of these activities after residency. That is, the vast majority of residents completing this rotation felt that they were likely to do at least one of these scholarly activities after graduation.

Further analyses among residents who reported that they were likely to initiate a research project after residency revealed that they rated the curriculum significantly higher on several objectives than residents who were not likely to initiate a research project. Residents who said that they were likely to initiate a research project after residency had stronger agreement that they learned more about data analysis (*p* < .05), felt more comfortable applying information from the medical literature to their practice (*p* < .05), and felt they were more likely to complete a scholarly project in the future (*p* < .05) than residents who did not think they were likely to initiate a research project after graduation. This could mean that residents who had already planned to initiate research projects after graduation from residency were more likely to benefit from the curriculum. These residents may have been more engaged with the training because it prepared them for their future goals. Alternatively, residents who learned more about data analysis and applying information from the medical literature to practice may have felt inspired and more capable of conducting their own research after residency.

## Discussion

An online, self-led research curriculum was developed and implemented in a family medicine residency and was well received by residents, who rated it highly in meeting curriculum objectives and in satisfaction. This suggests that an online, self-led curriculum can be a feasible and effective component of required residency training in scholarly activity.

The online format was convenient for residents. They were able to access the curriculum and readings from different locations and could refer back to the material any time they had research-related questions, both during and after residency. As the educational software provided immediate feedback on multiple-choice answers, residents were able to receive immediate reinforcement or correction. Additionally, residents were able to answer multiple-choice questions more than once, allowing multiple practices if the concept was not grasped on the initial trial.

The online modular format was utilized to align with residents' familiarity and reliance on online resources as primary information sources.^6^ Additionally, the self-directed format provided residents some flexibility in determining how they approached and completed the curriculum.^20^ The curriculum format supported the ACGME core competency of practice-based learning and improvement through residents' use of information technology to enhance learning and self-identification of strengths and weaknesses in research.^2^ The online format also ensured efficient use of faculty and resident time, such that faculty meetings could focus on residents' specific knowledge gaps and learning needs, rather than presenting all learning material uniformly. The LMS allowed faculty to view each attempt at answering multiple-choice questions, and thus, faculty could check resident understanding of concepts in those sections.

There were some challenges encountered with the implementation of this curriculum. First, residents who were not accustomed to managing their own time had difficulty achieving the curriculum goals by the deadlines. These residents would use the curriculum's protected time to finish other tasks, such as completing patient notes. Initial residents completing the curriculum had difficulty absorbing information from Module I due to attempts to read all assigned readings in one sitting. Additionally, we found that residents were often accustomed to skipping over instructions on computerized tasks and as a result would not know where to find needed information. After discovering these barriers, we implemented a section of the premeeting to address each barrier before initiation of the rotation.

We also found that a few residents needed a lot more help than others. As originally identified during development of the curriculum, residents had different levels of background knowledge and skills, which were now more easily identified through individual meetings with faculty. Three of the 18 residents needed additional time to meet the curriculum objectives and successfully complete the rotation. For each of these residents, the instructor and learner identified the unmet objectives and associated problem areas, for example, inability to determine study design or difficulty with calculation and interpretation of basic statistics. Once the problem areas were identified, the instructor directed the resident to the curriculum materials that corresponded to the problem areas; developed a plan for remediation, for example, a new time line for completion of the curriculum or walking the learner through sample statistical calculations; and scheduled a follow-up face-to-face meeting. Some residents required multiple additional meetings with faculty to improve understanding of critical analysis or to improve written communication. These residents were given extensions to complete the modules but did not receive additional protected time. While the additional meetings with these residents did require more resident and faculty time, the residents were found to benefit from the additional one-on-one training.

After implementation of this curriculum, many residents began their PGY-3 scholarly project earlier. Even among those residents who selected a PGY-3 scholarly project topic that was different from the one used for their rotation proposal, the PGY-3 project proposals were found to have more adequately developed research questions and better thought-out methods than in previous years. This was likely due to the practice of generating a written research protocol and receiving feedback on this protocol during the new curriculum.

While most residents agreed that curriculum objectives were met, some responded *neutral* on the Likert scale. This may have been because some of the residents already had significant research experience prior to residency and thus did not feel their skills individually were improved but did not disagree that the curriculum met this objective. Alternatively, as the survey was distributed at the end of the academic year, residents who had completed the curriculum towards the beginning of the year may not have recalled enough to agree or disagree with these questions.

This curriculum was implemented at one family medicine residency, so the sample size is small, and the generalizability of the findings may be limited. To ensure anonymity, the surveys were sent to all residents at once rather than when each completed the rotation. As a result, those who had completed the curriculum earlier may have had different recall of the curriculum than those who had completed the curriculum more recently. In the future, it would be beneficial to ask residents to submit evaluations to a neutral source at the end of the rotation and to collectively analyze the data at the end of the academic year to ensure that residents can give immediate feedback on the rotation and also maintain anonymity.

We would like to further investigate whether residency graduates who completed the rotation go on to participate in more scholarly work than those who did not complete the rotation. Future plans are to investigate how many residency graduates complete scholarly work within 5 years after graduation, compared to residents who graduated before the implementation of this curriculum.

The curriculum can be easily implemented at other residencies that have access to online educational software enabling information posting and administration of online quizzes with automated scoring. LMSs are already available and being used at many universities, and as a result, this curriculum could easily be uploaded to existing software without the need to learn or develop any new software. The content of this curriculum is not specific to family medicine and can be adapted, in full or in part, at residencies in other specialties. Once materials and tests are posted online, the curriculum is sustained by establishing a schedule for completion of rotation materials appropriate to the individual residency and by scheduling meetings with faculty during completion of the curriculum. Other residencies may choose to replace some of the readings with material on topics more closely aligned with their specialty.

## Appendices

A. Rotation Overview.pdf
B. Additional Questions.pdf
C. Questions for Module II.docx
D. Sample Answers for Module II.docx All appendices are peer reviewed as integral parts of the Original Publication.

## References

[1] Accreditation Council for Graduate Medical Education. Specialty-specific references for DIOs: resident/fellow scholarly activity. Accreditation Council for Graduate Medical Education website. http://www.acgme.org/Portals/0/PDFs/Specialty-specific%20Requirement%20Topics/DIO-Scholarly_Activity_Resident-Fellow.pdf?ver=2016-04-04-150606-760. Published February 2015. Accessed April 17, 2015.

[2] Accreditation Council for Graduate Medical Education. ACGME program requirements for graduate medical education in family medicine. Accreditation Council for Graduate Medical Education website. http://www.acgme.org/acgmeweb/Portals/0/PFAssets/ProgramRequirements/120_family_medicine_07012014.pdf. Updated 2013. Accessed January 14, 2015.

[3] Crawford P, Seehusen D Scholarly activity in family medicine residency programs: a national survey. Fam Med. 2011;43(5):311–317. 21557099

[4] Simasek M, Ballard SL, Phelps P, et al. Meeting resident scholarly activity requirements through a longitudinal quality improvement curriculum. J Grad Med Educ. 2015;7(1):86–90. https://doi.org/10.4300/JGME-D-14-00360.1 26217429 10.4300/JGME-D-14-00360.1 PMC4507935

[5] Hoedebecke K, Rerucha C, Runser L Increase in residency scholarly activity as a result of resident-led initiative. Fam Med. 2014;46(4):288–290. 24788425

[6] Duran-Nelson A, Gladding S, Beattie J, Nixon LJ Should we Google it? Resource use by internal medicine residents for point-of-care clinical decision making. Acad Med. 2013;88(6):788–794. https://doi.org/10.1097/ACM.0b013e31828ffdb7 23619072 10.1097/ACM.0b013e31828ffdb7

[7] Oxman AD, Sackett DL, Guyatt GH, et al. Users' guides to the medical literature: I. How to get started. JAMA. 1993;270(17):2093–2095. https://doi.org/10.1001/jama.1993.03510170083036 8411577

[8] Guyatt GH, Sackett DL, Cook DJ, et al. Users' guides to the medical literature: II. How to use an article about therapy or prevention. A. Are the results of the study valid? JAMA. 1993;270(21):2598–2601. https://doi.org/10.1001/jama.1993.03510210084032 8230645 10.1001/jama.270.21.2598

[9] Guyatt GH, Sackett DL, Cook DJ, et al. Users' guides to the medical literature: II. How to use an article about therapy or prevention. B. What were the results and will they help me in caring for my patients? JAMA. 1994;271(1):59–63. https://doi.org/10.1001/jama.1994.03510250075039 8258890 10.1001/jama.271.1.59

[10] Jaeschke R, Guyatt G, Sackett DL, et al. Users' guides to the medical literature: III. How to use an article about a diagnostic test. A. Are the results of the study valid? JAMA. 1994;271(5):389–391. https://doi.org/10.1001/jama.1994.03510290071040 8283589 10.1001/jama.271.5.389

[11] Jaeschke R, Guyatt GH, Sackett DL, et al. Users' guides to the medical literature: III. How to use an article about a diagnostic test. B. What are the results and will they help me in caring for my patients? JAMA. 1994;271(9):703–707. https://doi.org/10.1001/jama.1994.03510330081039 8309035 10.1001/jama.271.9.703

[12] Laupacis A, Wells G, Richardson WS, et al. Users' guides to the medical literature: V. How to use an article about prognosis. JAMA. 1994;272(3):234–237. https://doi.org/10.1001/jama.1994.03520030076032 8022043 10.1001/jama.272.3.234

[13] Bewick V, Cheek L, Ball J Statistics review 13: receiver operating characteristic curves. Crit Care. 2004;8(6):508–512. https://doi.org/10.1186/cc3000 15566624 10.1186/cc3000 PMC1065080

[14] Matthews JR, Matthews RW Follow standard structure. In: Successful Scientific Writing: A Step-by-Step Guide for the Biological and Medical Sciences. 3rd ed. Cambridge, England: Cambridge University Press; 2008:42–48.

[15] Bem DJ Writing the empirical journal article. In: Darley JM, Zanna MP, Roediger HL III, eds. The Compleat Academic: A Career Guide. 2nd ed. Washington, DC: American Psychological Association; 2004:185–219.

[16] Lynn R Race differences in sexual behavior and their demographic implications. Popul Environ. 2000;22(1):73–81. https://doi.org/10.1023/A:1006633632359

[17] MacMahon B, Yen S, Trichopoulos D, Warren K, Nardi G Coffee and cancer of the pancreas. N Engl J Med. 1981;304(11):630–633. https://doi.org/10.1056/NEJM198103123041102 7453739 10.1056/NEJM198103123041102

[18] Kulasingam SL, Hughes JP, Kiviat NB, et al. Evaluation of human papillomavirus testing in primary screening for cervical abnormalities: comparison of sensitivity, specificity, and frequency of referral. JAMA. 2002;288(14):1749–1757. https://doi.org/10.1001/jama.288.14.1749 12365959 10.1001/jama.288.14.1749

[19] Ferreira FL, Bota DP, Bross A, Mélot C, Vincent J-L Serial evaluation of the SOFA score to predict outcome in critically ill patients. JAMA. 2001;286(14):1754–1758. https://doi.org/10.1001/jama.286.14.1754 11594901 10.1001/jama.286.14.1754

[20] Lebensohn P, Kligler B, Dodds S, et al. Integrative medicine in residency education: developing competency through online curriculum training. J Grad Med Educ. 2012;4(1):76–82. https://doi.org/10.4300/JGME-04-01-30 23451312 10.4300/JGME-04-01-30 PMC3312539

