# Supplementary material for: An Online, Self-Directed Curriculum of Core Research Concepts and Skills
Source: MedEdPORTAL. 2018 Jul 27;14:10732. doi: 10.15766/mep_2374-8265.10732 (PMC6346278; doi:10.15766/mep_2374-8265.10732)
Supplement: Supplementary file 1 — A. Rotation Overview.pdf B. Additional Questions.pdf C. Questions for Module II.docx D. Sample Answers for Module II.docx [file mep-14-10732-s001.zip › A._Rotation_Overview.pdf]

Research Curriculum

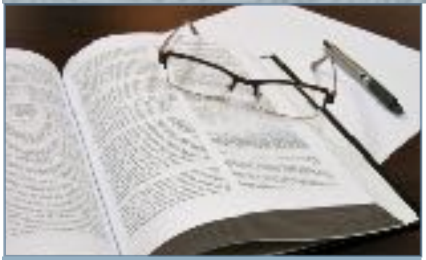

CONTENTS

PAGE 1
MODULES I-III
You will use concepts learned in Module I to complete tasks in Modules II - V.

PAGE 2
MODULE IV-V
Includes information on using the campus library and useful databases.

PAGE 3
SCHEDULE AND FAQ
A recommended schedule for completing the rotation and frequently asked questions.

PAGE 4
COMPLETING THE ROTATION
Includes a checklist to ensure successful completion of all modules.

Rotation Overview

The two-week research rotation consists of five modules:

Week 1:

- ✦ Module I: Research Methods and Data Analysis
- ✦ Module II: Article Review
- ✦ Module III: Board Review

Week 2:

- ✦ Module IV: Literature Search
- ✦ Module V: Literature Review and Proposal

Module Descriptions

Module I: Research Methods and Data Analysis

Module I is a learning module. In this module, you will read several articles and chapters that will teach you about research methods, data analysis, and writing. You will then use information from these readings for the other Modules, your senior project, and any future projects.

All material for this module is posted on Blackboard.

What to complete for Module I:

- ✦ Read *Users' Guides to the Medical Literature I-III* (posted as 1 document)
- ✦ Read *Users Guides to the Medical Literature V*
- ✦ Read *Stats Review* article
- ✦ Read *Additional Questions*
- ✦ Read *Standard Structure* article
- ✦ Read *Writing the Empirical Journal*

Module II: Article Review

Module II is an opportunity for you to practice applying what you have learned in Module I. You will use questions from the *Users' Guides* articles and the *Additional Questions* reading to critique Articles 1 and 2. You will use information from the *Stats Review* article to interpret Articles 3 and 4. It is recommended that you meet with a faculty member to discuss these 4 articles before completing the tests.

What to complete for Module II:

- ✦ Read Research Article 1:*Race Differences in Sexual Behavior*
- ✦ Complete Article 1 questions
- ✦ Read Research Article 2: *Coffee and Cancer of the Pancreas*
- ✦ Complete Article 2 questions
- ✦ Read Research Article 3: *Evaluation of HPV Testing in Primary Screening for Cervical Abnormalities*
- ✦ Complete Article 3 questions
- ✦ Read Research Article 4: *Serial Evaluation of the SOFA Score to Predict Outcome in Critically Ill Patients*
- ✦ Complete Article 4 questions

Module III: Board Review

The goal for this section is for you to learn how to answer questions that you might see on the boards. You will be using information from Module I to answer board review questions. The key for this module is to learn, not to get all the right answers the first time. Since trying questions first, making mistakes, and then looking up the answers afterward to has been found to help people do better on the exam, it is recommended that you use this method.

What to complete for Module III:

- ✦ Download Board Review Questions from Blackboard

- Answer the questions in the Board Review Test on Blackboard. You will receive feedback at the end of the test.
- Download the Board Review Answers to review reasons for the correct answers

You will be allowed multiple attempts on this test on Blackboard. It is recommended that you take the test, review the correct answers as above, then wait several days and take the test again. As you may be aware, research on memory has found that reviewing material twice, with a lag time in between that includes sleep, increases encoding of information into long term memory. So taking the test a second time will solidify your knowledge, and increase your score on the test.

Module 4: Literature Search

A topic for scholarly project should be selected and reviewed with a faculty member. You will then conduct a literature search to determine what has already been done on that topic. You can use information from Module I on narrowing down your search terms.

Literature searches should include use of databases for scholarly articles. Examples of these include:

- PubMed
- Medline
- PsycInfo
- PsycArticles
- Google Scholar

or other databases found through the campus website. These databases can be found by going to:
[insert your residency library resource here].

Literature searches may also include *articles or reports* that are posted on foundation or organization websites.

For example, the Sexually Transmitted Disease Surveillance 2007 Supplement: Syphilis Surveillance Report is available through the CDC website at: http://www.cdc.gov/std/Syphilis2007/Syphilis2007Short.pdf
You can tell that this is an article or report posted to the website because you can clearly identify the author (Department of Health and Human Services, Centers for Disease Control and Prevention) and the date of publication (March 2009).

Literature searches do *not* include information that is posted on a website, even if it is a reputable site. Examples of inappropriate sources of information include:

Medical information websites, such as this Syphilis information posted on WebMD: http://www.webmd.com/sexual-conditions/guide/syphilis

Up-to-Date

Google searches

Wikipedia (looking up medical or factual information on Wikipedia is NEVER appropriate. It should be used only to look up popular opinions or entertainment.)

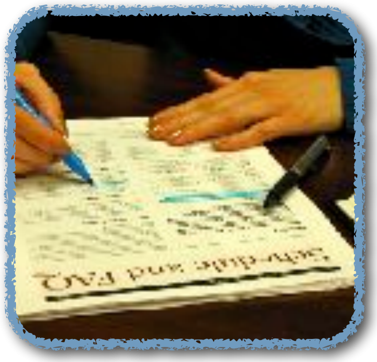

It may be helpful to organize your articles using a reference management system. One of these systems, Refworks, is available for free through the campus library website. Video tutorials are available to teach you to use the software. Use of a reference management system is not required for this rotation. However, you may like to use it because you can automatically save references and PDFs from database searches, and it will create and format bibliographies for you, which can be time consuming.

Module V: Literature Review and Proposal

A written literature review and project proposal is due on the last day of the research rotation. This is part of your Interpersonal and Communication Skills Core Competency. Most physicians have not developed adequate writing skills, yet much of communication in medicine is done through writing. This component will help you to improve your writing skills and will help you to organize and plan your senior project. You should use information from the Writing and Publishing section of Module I.

The first section is your literature review. This is your Introduction section. This section should summarize findings in the articles that you read during your literature search. You should cite a minimum of 10 articles. At least two of these articles should be experimental studies. The literature review should address the following questions:

- What is your topic?
- Why is it important?
- What has been found so far?
- What is still unknown?

For quality improvement projects, it may be necessary to gather additional information that is not available through literature searches. For example, a family medicine residency program created a protocol to help residents struggling with medical knowledge improve their performance. They implemented the protocol and found favorable results. They then presented this protocol and their findings at the annual meeting of the Society for Teachers of Family Medicine and posted their presentation on www.fmdrl.org. Although this is not an article in the literature, it would be appropriate to use this Powerpoint as a resource, describing what they tried and found, and cite it as a model for your proposed QI project.

The Proposal is where you articulate the methods of what you plan to do. This should be in some detail. The proposal should include:

- What you plan to do.
- What you plan to measure.
- How you plan to measure it.
- How your terms will be defined.
- Who your population will be.

Schedule and FAQ

Schedule

Much of this rotation is self-led. This means that you will be challenged to self-motivate to complete work, and to schedule your own time. This is part of your ACGME Core Competencies of Practice Based Learning and Improvement, and Professionalism. While most of your residency is scheduled for you, this is your opportunity to demonstrate your ability to independently manage your own time.

Recommended Schedule

It is recommended that you schedule a 15-minute meeting with a faculty member before your research rotation is scheduled to begin. This will allow you to review the requirements of this rotation and ask any questions you might have.

It is recommended that you complete Sections I, II, and III of the curriculum in your **first week**. You should also be thinking of a scholarly project topic during the first week so that you can promptly begin Section IV during the second week with your topic already selected.

It is required that you meet with faculty twice for this rotation. The first meeting should occur during the first week. You will be discussing the articles in Modules I and II. Be prepared to ask questions. This discussion can be utilized to ensure that you are on the right track before completing Article Review tests.

The second meeting with faculty should occur at the end of the rotation. At this time you should be prepared to turn in a draft of your Literature Review and Proposal, and to receive feedback.

Can we work in pairs or groups?

You are permitted to work together with your colleagues to complete your senior project. However, you will still each be responsible for completing a literature and research proposal. For example, a pair of you may wish to do a quality improvement project to improve asthma management in the clinic. One of the pair could complete the literature review on asthma and the effects of poor management. The other member of the pair could complete the literature review on methods used to improve management, and what quality improvement methods other clinics have implemented.

How do I best use the library?

You can work from the library, or from any other location while completing the research curriculum.

Can I publish my project?

This curriculum is designed to make it easy for you to publish your senior project. The Literature Review and Proposal will be the draft of your Introduction and Methods sections. You will have to update them after you complete the project, to specify the methods that were actually used. Upon completing your senior project, you will just have to write up the Results and Discussion sections.

How long should my Literature Review and Proposal be?

This will depend on your topic, and the complexity of your research question. You may want to look in journals that publish articles you cite to see how long their introduction and methods sections are. This is good practice as those journals have demonstrated interest in publishing articles on that topic, and you may be able to publish there. For the purposes of this curriculum you should be aiming for at least 1000-1500 words. If it looks like you are getting a 10-page intro, either write more concisely, or narrow your topic.

What are the objectives of the curriculum?

By the end of this curriculum, learners will be able to:

1. Apply critical reading skills to determine study validity and interpret study results in the medical literature
2. Demonstrate ability to critically evaluate research and medical literature
3. Identify strengths and weaknesses in study designs
4. Practice calculating and interpreting basic statistics
5. Apply basic statistical concepts to interpreting the medical literature
6. Synthesize knowledge gained in the curriculum to develop a research question and methods to evaluate the question
7. Develop writing skills through preparation of a project proposal

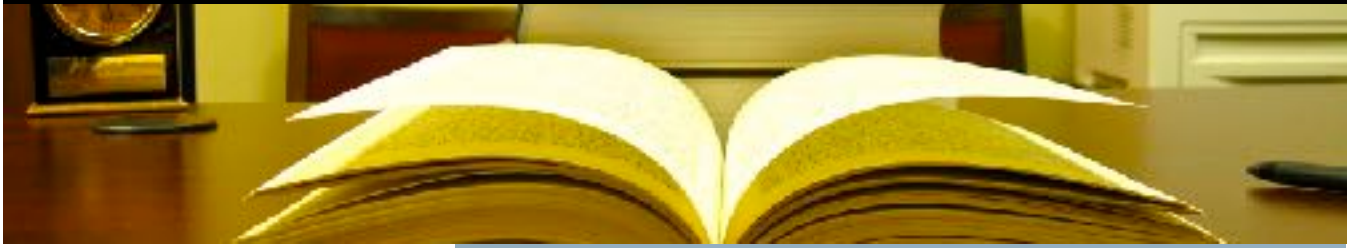

Do your best!

Completing the Rotation

Flexible time is rare in residency training. As a result, it may be difficult to motivate yourself to work, and you may feel tempted to put forth partial effort. Please note that successful completion of this rotation is contingent on *adequate* achievement in all sections. That is, your work during this rotation will be judged based on *quality*, not solely on whether or not the task was completed.

Failure to adequately complete all sections of this curriculum will result in failure of the rotation. If the rotation is failed, you will be responsible for repeating all of the curriculum components. This includes selecting a new topic for Literature Search, Review, and Proposal. Due dates for completion of the the curriculum components will be determined by the Research Rotation Faculty. The time allotted for the Research Rotation will not be repeated, and therefore work will be completed on top of other rotations. Additionally, test materials must be completed independently, and without the help of peers. However, it is permissible to discuss posted articles with faculty prior to completing the Blackboard assignments on them.

CHECKLIST

The following items should be completed by the end of the rotation.

MODULE I

- ☐ Read *Users' Guides to the Medical Literature I-III*
- ☐ Read *Users' Guides to the Medical Literature V*
- ☐ Read *Stats Review* article
- ☐ Read *Additional Questions*
- ☐ Read *Standard Structure* article
- ☐ Read *Writing the Empirical Journal Article*

MODULE II

- ☐ Read Race Differences in Sexual Behavior
- ☐ Read Coffee and Cancer of the Pancreas
- ☐ Read *Evaluation of Human Papillomavirus Testing in Primary Screening for Cervical Abnormalities: Comparison of Sensitivity, Specificity, and Frequency of Referral*
- ☐ Read *Serial Evaluation of the SOFA Score to Predict Outcome in Critically Ill Patients*
- ☐ Meet with faculty member to discuss articles
- ☐ Complete Questions for the four Module II articles

MODULE III

- ☐ Take and review Board Review Questions

MODULE IV

- ☐ Complete Literature Search

MODULE V

- ☐ Write Literature Review and Proposal
- ☐ Submit Literature Review and Proposal to faculty member
- ☐ Meet with faculty member to discuss literature review
- ☐ Forward corrected draft of Literature Review and Proposal to selected Research Advisor
- ☐ *Optional:* Read information in the Additional Readings and Resources Folder
