# Supplementary material for: An Online, Self-Directed Curriculum of Core Research Concepts and Skills
Source: MedEdPORTAL. 2018 Jul 27;14:10732. doi: 10.15766/mep_2374-8265.10732 (PMC6346278; doi:10.15766/mep_2374-8265.10732)
Supplement: Supplementary file 1 — A. Rotation Overview.pdf B. Additional Questions.pdf C. Questions for Module II.docx D. Sample Answers for Module II.docx [file mep-14-10732-s001.zip › B._Additional_Questions.pdf]

# Additional Questions for Reading Articles

## When determining the validity of a study, ask yourself these questions

### *How are the variables defined?*

To conduct any research study (or quality improvement project) the variables must be defined in a way that can be measured. This is called an ***operational definition.*** Everything that we study starts out as an abstract concept, until we define it in a way that can be categorized or measured.

For example, a study is investigating the effect of Drug A on dementia severity. Assuming that the definition of Drug A has already been described in another publication, this study should specify the definition of dementia. How are the participants going to be categorized as having dementia, or not? Different studies may use different operational definitions for dementia, which can lead to different results. For example, some studies may only include participants with Alzheimer's Type Dementia, while others include multiple types. Some studies may include a Mini-Mental Status Exam score of less than 18 in their definition, while other may include a score of less than 26.

Similarly, this study should specify the definition that will be used to determine severity Severity could be defined as a score on a cognitive test, tissue damage visible on imaging studies, or level of functioning.

### *Does the measurement match the definition?*

Using the dementia study above, assume the authors have defined dementia severity as the impairment of patient functioning. They decide to determine whether participants have each of 5 symptoms of dementia (i.e. memory impairment, aphasia, apraxia, agnosia, and executive function impairment). They count the number of symptoms each participant has and assign a value of 1 to 5, indicating the total number of symptoms. Patients with a score of 5, that is all 5 symptoms, have the most impaired functioning.

In this case, the measurement is not a good match with the definition. It has poor construct validity. The term ***construct validity*** refers to whether what you say you are measuring is actually what you are measuring. The authors of this study say they are measuring functioning, but they are actually measuring number of symptoms. Number of symptoms may contribute to functioning, but they are not necessarily the same thing.

A measure that might have better construct validity for patient functioning could be determining the number of Activities of Daily Living that the participant can complete independently.

### *Do the conclusions match the data?*

Researchers are susceptible to bias. As a result, some may interpret their data in ways that support their theory or hypothesis. The scientists conducting the study on the effect of Drug A on dementia severity may believe that Drug A is very effective. In their experiment, they had 200 patients, half taking Drug A and half taking placebo. The placebo group had no change in dementia severity during the study, while more than half (60%) of the treatment group had improvements in their severity scores. The authors conclude this supports the drugs efficacy.

Upon closer inspection of the data, you might find that the 60% who improved had an average improvement of 5 points on their severity scores. However, the 40% who did not improve had an average decline of 40 points. This additional information should affect whether you decide to accept the authors' conclusions, and whether you start prescribing Drug A to your patients.

As you read articles, and as you complete your own projects, remember "If you torture data sufficiently, it will confess to almost anything"-Dr. Fred Menger, chemistry professor.

### *How many other studies have similar findings?*

People often forget that statistical significance cannot "prove" anything. A p-value is just the probability that the researchers could have gotten their results by chance. A p-value of .05 means that there is a 5% chance that any differences in groups occurred by chance. This means that many published studies obtained significant results by chance and that there is no real relationship.

Additionally, journals often favor studies with significant results when deciding which studies to publish. In the dementia study, there may be 20 other studies that have been conducted that have found no effect of Drug A on dementia severity which may not have been published. Be cautious of studies that stand alone and have not been replicated. Have more confidence in conclusions with consistent support across studies and authors.
