# Supplementary material for: An Online, Self-Directed Curriculum of Core Research Concepts and Skills
Source: MedEdPORTAL. 2018 Jul 27;14:10732. doi: 10.15766/mep_2374-8265.10732 (PMC6346278; doi:10.15766/mep_2374-8265.10732)
Supplement: Supplementary file 1 — A. Rotation Overview.pdf B. Additional Questions.pdf C. Questions for Module II.docx D. Sample Answers for Module II.docx [file mep-14-10732-s001.zip › C._Questions_for_Module_II.docx]

**Questions for Articles in Module II**

Questions for Article 1: Race Differences in Sexual Behavior

*All questions for this article are essay format.*

1. The introduction section describes the background and theory behind this study.  The methods section details how the hypothesis/theory will be tested.  Describe flaws in this study's introduction and methods sections.
2. The results section lists a number of significant differences.  Describe how the numbers and measures used may be misleading.
3. The Discussion section conclude that three of the four "predictions were confirmed."  Describe flaws in the conclusions and explanations in the discussion section.

Question for Article 2: Coffee and Cancer of the Pancreas

*Question for this article is essay format.*

1. This study concludes that there is a strong association between pancreatic cancer and coffee consumption.  However, the American Cancer Society no longer considers coffee as a significant risk factor for pancreatic cancer. What are some factors that may lead you to question the conclusions of this study?

Questions for Article 3: Evaluation of Human Papillomavirus Testing in Primary Screening for Cervical Abnormalities

*Questions for this article are multiple choice.*

The following questions will use data from the Evaluation of HPV Testing in Primary Screening for Cervical Abnormalities article. Since the article uses a multilayer design, we will only look at one of the comparisons here. We will also only look at uncorrected values for these questions, so you can ignore the correction equations presented in the article.

For the following questions, we will use PCR as the "gold standard" to determine whether someone is HPV+. Cytology of > ASCUS is the test.  The article states that 380 women had > Ascus and were HPV+ by PCR; 377 had > ASCUS and were HPV- by PCR; 533 had normal cytology and were HPV+ by PCR; and 2631 had normal cytology and were HPV- (for a total of 3921 women).  Using these values, and what you learned from the Stats Review article, answer the following.

1. Which of the following will be affected by the prevalence of HPV?
   1. Positive Predictive Value
   2. Likelihood Ratio
   3. Sensitivity
   4. Specificity
2. What is the sensitivity of using > ASCUS to test for HPV status?
   1. 0.30
   2. 0.42
   3. 0.68
   4. 0.83
   5. 0.87
3. What is the specificity of using > ASCUS to determine HPV status?
   1. 0.30
   2. 0.42
   3. 0.68
   4. 0.83
   5. 0.87
4. A woman’s cytology is > ASCUS. Using the data from the instructions above, what is the probability that she actually has HPV?
   1. 32.1%
   2. 44.3%
   3. 50.2%
   4. 73.8%
   5. 91.7%

Questions for Article 4: Serial Evaluation of the SOFA Score

*Questions for this article are multiple choice.*

Patients who are admitted to an ICU for more than 24 hours have a SOFA score calculated at admission and every 48 hours until discharge.  Using the ROC curves in Figure 2 of this article, answer the following questions.

1. If you wanted to make the best guess as to whether an ICU patient was going to die, which of the following would you use?
   1. Initial SOFA Score
   2. Mean SOFA Score
   3. Highest SOFA Score
   4. Total SOFA Score
   5. SOFA Score at 48 h
   6. SOFA Score at 96 h
   7. Change-SOFA Score 48-0 h
   8. Change-SOFA Score 96-0 h
2. Which of the following is the worst at predicting patient mortality?
   1. Initial SOFA Score
   2. Highest SOFA Score
   3. Total SOFA Score
   4. Change-SOFA score 48-0 h
   5. Change-SOFA Score 96-0 h
