# Supplementary material for: An Online, Self-Directed Curriculum of Core Research Concepts and Skills
Source: MedEdPORTAL. 2018 Jul 27;14:10732. doi: 10.15766/mep_2374-8265.10732 (PMC6346278; doi:10.15766/mep_2374-8265.10732)
Supplement: Supplementary file 1 — A. Rotation Overview.pdf B. Additional Questions.pdf C. Questions for Module II.docx D. Sample Answers for Module II.docx [file mep-14-10732-s001.zip › D._Sample_Answers_for_Module_II.docx]

# Sample Answers for Module II

*Note: Other responses for Articles 1 and 2 may also be correct.

## Article 1: Race Differences in Sexual Behavior

### Question 1: The introduction section describes the background and theory behind this study. The methods section details how the hypothesis/theory will be tested. Describe flaws in this study's introduction and methods sections.

*Introduction*

- r-K theory is a description of differences between phyla, and thus using it to try to describe a within-species difference does not apply. All primates are at the K end of the continuum, and are incapable of producing the number of offspring that other species can produce (e.g. hundreds to millions of offspring)
- Race is a social construct, and the use of three category groups does not apply to racial categories or identification.
- Applying evolutionary theories and categorization that describe physical differences to describing behaviors instead is an inappropriate use of those theories. Additionally, there is no description or control for any social factors in those behaviors.
- Articles referenced in the introduction are sparse.
- Parts of the introduction are not referenced and appear to be assumptions based on the already faulty theory. These assumptions are also expanded with little background or support.
- The hypothesis is not related to the background theory because number of partners and frequency of intercourse do not determine the number of offspring, or the care dedicated to rearing the offspring.

*Methods*

- Participants are all from the United States, with no sampling from East Asia, Europe, South Asia, or sub-Saharan Africa, which is what the author proposes is the basis of racial categorization
- Participants are asked to self-identify race, which is not consistent with the definitions set forth in the introduction
- The methods do not state that participants were limited to those of reproductive age, which would be essential if studying a reproductive strategy
- Age and relationship status were not assessed, despite asking about number of sexual partners in the last 5 years, and frequency of intercourse over the last year. It is possible that someone who is 50 and has been married for 20 years may have different sexual behaviors than someone who is 25 and single.
- People were only categorized as “black” or “white,” despite the original hypothesis also including people of Asian descent.
- People in hospitals were excluded, which can exclude the people who are actually having babies.
- There is not a sufficient description of why people were excluded.
- There can be a strong recall bias when asking about behaviors over the past year, or past 5 years. It may be hard for people to accurately recall frequency of intercourse over the past 52 weeks, and they may rely on more readily available information (e.g. frequency of intercourse over the past month)
- Frequency of intercourse can change across a year.
- There is no explanation as to why number of partners across 5 years was selected.
- There is no indication whether sexual activity was limited to heterosexual sex. Same sex sexual behavior does not lead to offspring, which is the base of this article
- The questions are on a sensitive topic, and methods do not describe factors that impact responses to sensitive questions (e.g. how the questions were asked, anonymity, etc.)
- Factors such as socioeconomic status and health may impact sexual behavior, but these were not controlled for
- There is no data on number of offspring or care provided to offspring.
- It is not clear if 1500 people is a large enough sample for the number of comparisons.

### Question 2: The results section lists a number of significant differences. Describe how the numbers and measures used may be misleading.

- It is unclear what the N represents in the table, since the methods section states that only 1500 people were surveyed.
- The differences found were statistically significant, but not meaningfully significant. That is, there is not a meaningful difference between having sex 3.1 times a week and 3.5 times a week. Similarly, there is not a meaningful difference between 1.3 sexual partners and 1.6 sexual partners. With standard deviations, the range of responses are highly overlapped between groups.
- Rushton’s theory does not differentiate between male and female behavior. Thus, it is not clear why the author chose to separate findings based on sex. It is possible that the comparisons would not have been significant without this breakdown.

Note: many residents may respond that there is a large difference in the number of white participants and black participants. This, however, is not necessarily a flaw. When a sample selected to be representative of the population, it is expected that the distribution of participants in the sample would mirror the distribution in the general population.

### Question 3: The Discussion section concludes that three of the four "predictions were confirmed." Describe flaws in the conclusions and explanations in the discussion section.

- Although the author’s hypotheses were not supported by all comparisons in the results section, rather than revising the hypotheses, the author tries instead to explain why the data may have been inaccurate (e.g. people do not necessarily report their sexual behavior accurately.) However, if this statement is true, it applies to the whole sample, not just to the comparison without significant findings.
- The author’s explanation that the frequency of intercourse may have been non-significant because of preference for partner based on race (i.e. fewer partners choosing black females compared to white females) actually would apply to number of partners, not to the frequency of intercourse.
- Many of the conclusions are not based on the data presented
- The section on Sub-Saharan Africa is irrelevant

## Article 2: Coffee and Cancer of the Pancreas

### Question 1: This study concludes that there is a strong association between pancreatic cancer and coffee consumption.  However, the American Cancer Society no longer considers coffee as a significant risk factor for pancreatic cancer. What are some factors that may lead you to question the conclusions of this study?

- p < .05 means that there is less than 5% chance that the findings were by chance alone. This article was the only article that has found this result. Previous and subsequent studies have not been able to replicate it. If a study is in a reputable journal, is peer-reviewed, and a well-designed study, it still needs to be replicated to determine if the results were found by chance.
- Other potential limitations include:
  - Exclusion of highly ill patients
  - Exclusion of non-white participants
  - Exclusion of other diagnoses that may be more representative of the clinical population
  - Age limitations
  - Geographic limitations
  - Study is not randomized
  - Convenience sampling
  - Interviewer not blinded
  - Patient responses may be skewed based on diagnosis (those with pancreatic cancer may respond to questions differently than those in the control group without a cancer diagnosis.

## Article 3: Evaluation of Human Papillomavirus Testing in Primary Screening for Cervical Abnormalities

| Test | Outcome | |  |
| --- | --- | --- | --- |
|  | PCR+ | PCR- | Total |
| >ASCUS | 380 | 377 | 757 |
| <ASCUS | 533 | 2631 | 3164 |
| Total | 913 | 3008 | 3921 |

1. Which of the following will be affected by the prevalence of HPV?
   1. **Positive Predictive Value**
   2. Likelihood Ratio
   3. Sensitivity
   4. Specificity
2. What is the sensitivity of using > ASCUS to test for HPV status?
   1. 0.30
   2. **0.42**
   3. 0.68
   4. 0.83
   5. 0.87
3. What is the specificity of using > ASCUS to determine HPV status?
   1. 0.30
   2. 0.42
   3. 0.68
   4. 0.83
   5. **0.87**
4. A woman’s cytology is > ASCUS. Using the data from the instructions above, what is the probability that she actually has HPV?
   1. 32.1%
   2. 44.3%
   3. **50.2%**
   4. 73.8%
   5. 91.7%

## Article 4: Serial Evaluation of the SOFA Score to Predict Outcome in Critically Ill Patients

1. If you wanted to make the best guess as to whether an ICU patient was going to die, which of the following would you use?
   1. Initial SOFA Score
   2. Mean SOFA Score
   3. **Highest SOFA Score**
   4. Total SOFA Score
   5. SOFA Score at 48 h
   6. SOFA Score at 96 h
   7. Change-SOFA Score 48-0 h
   8. Change-SOFA Score 96-0 h
2. Which of the following is the worst at predicting patient mortality?
   1. Initial SOFA Score
   2. Highest SOFA Score
   3. Total SOFA Score
   4. Change-SOFA score 48-0 h
   5. **Change-SOFA Score 96-0 h**
